# Measuring preferences for analgesic treatment for cancer pain: How do African-Americans and Whites perform on choice-based conjoint (CBC) analysis experiments?

**DOI:** 10.1186/1472-6947-13-118

**Published:** 2013-10-18

**Authors:** Salimah H Meghani, Jesse Chittams, Alexandra L Hanlon, Joseph Curry

**Affiliations:** 1 Department of Biobehavioral Health Sciences, NewCourtland Center for Transitions & Health, University of Pennsylvania School of Nursing, Claire M. Fagin Hall, 418 Curie Boulevard, Room 337, 19104-4217 Philadelphia, PA, USA; 2 University of Pennsylvania School of Nursing, 418 Curie Blvd. Room 492, 19104 Philadelphia, PA, USA; 3 Sawtooth Technologies, Inc., 1500 Skokie Boulevard, 60062 Northbrook, IL, USA

## Abstract

**Background:**

Conjoint Analysis (CA) can serve as an important tool to study health disparities and unique factors underlying decision-making in diverse subgroups. However, methodological advancements are needed in exploiting this application of CA. We compared the internal and external predictive validity and inter-temporal stability of Choice-based-Conjoint (CBC) analysis between African-Americans and Whites in the clinical context of preferences for analgesic treatment for cancer pain.

**Methods:**

We conducted a prospective study with repeated-measures at two time-points (T1 = baseline; T2 = 3-months). African-Americans (n = 102); and Whites (n = 139) with cancer-related pain were recruited from outpatient oncology clinics in Philadelphia. Informed by pilot work, a computer-assisted CBC experiment was developed using 5 attributes of analgesic treatment: type of analgesic; expected pain relief; type of side-effects; severity of side-effects; and out-of-pocket cost. The design included 2 choice alternatives, 12 random tasks, 2 holdout tasks, and maximum of 6 levels per attribute. The internal and external predictive validity of CBC was estimated using Root Likelihood (RLH) and Mean Absolute Error (MAE), respectively. Inter-temporal stability was assessed using Cohen’s kappa.

**Results:**

Whites predominantly traded based on “pain relief” whereas African-Americans traded based on “type of side-effects”. At both time-points, the internal validity (RLH) was slightly higher for Whites than for African-Americans. The RLH for African-Americans improved at T2, possibly due to the learning effect. Lexicographic (dominant) behavior was observed in 29% of choice datasets; Whites were more likely than African-Americans to engage in a lexicographic behavior (60% vs. 40%). External validity (MAE) was slightly better for African-Americans than for Whites at both time-points (MAE: T1 = 3.04% for African-Americans and 4.02% for Whites; T2 = 8.04% for African-Americans; 10.24% for Whites). At T2, the MAE increased for both groups possibly reflecting an increase in the complexity of pain treatment decision-making based on *expectations* (T1) as opposed to *reality* (T2). The inter-temporal stability was fair for CBC attributes between T1 and T2 (kappa = 0.28, 95% CI: 0.24-0.32) and was *not* predicted by demographics including race.

**Conclusions:**

While we found slight group differences, overall the internal and external predictive validity of CBC was comparable between African-Americans and Whites. We discuss some areas to investigate and improve internal and external predictive validity of CBC experiments.

## Background

The healthcare and funding structures in the U.S. have recently placed an unprecedented emphasis on the role of patients’ perspectives in healthcare outcomes [1]. These directions necessitate understanding of techniques that improve assessment of patient-reported outcomes including the important intermediary outcomes of preferences and decision-making.

Conjoint Analysis (CA) is a valuation technique grounded in random utility theory [2] and mathematical psychology [3] to understand what people value and what drives them to choose one set of alternatives over another when faced with competing choices [4]. There has been a rapid increase in the application of CA in the health related research over the past decade [5]. The main premise of CA is that individuals derive utility from the properties or characteristics of a good rather than the good itself [6]. Thus, the utility or desirability of any good (e.g. health services or treatment alternatives) can be described based on the value of its separate, yet, conjoined parts. These separate but conjoined parts are termed “attributes” each with multiple “levels” [7]. By asking individuals to make trade-offs between an important but limited number of attributes, a unique set of values (“preference weights” or “part-worth utilities”) can be derived. These preference weights are results of modeling the underlying latent utility function such that a higher preference weight represents a higher value an individual assigns to that attribute [7]. The attributes can then be compared to one another to ascertain the “relative importance” or the percentage of total variance in preferences that each attribute explains.

The relative importance an individual associates with an attribute is also expected to vary based on an individual’s background (e.g., demographics) or clinical factors (e.g., expectations or past experiences with treatments) [8]. As may be evident, CA can serve as an important clinical and research tool to understand racial and ethnic disparities and what unique factors may underlie decision-making in diverse patient groups. This application of CA is beginning to be exploited in health literature [9,10], and no studies to our knowledge, have compared the predictive validity and temporal stability of CA techniques among diverse subgroups of minorities.

Cancer pain treatment decisions are preference-sensitive [11] and clinically important racial disparities have been reported in preferences and adherence to analgesics for cancer pain [12-17]. CA can offer an important avenue to understand heuristics underlying cancer pain treatment decisions. However, the use of CA techniques to understand clinical disparities in preferences and decision-making requires addressing methodological issues including validity of this method in diverse patient populations. In this paper, we present one empirical example of comparing validity of conjoint analysis in diverse subgroups. More specifically, we compare the internal and external predictive validity and inter-temporal stability of Choice-based Conjoint (CBC) analysis between African-Americans and Whites in the clinical context of understanding their preferences for analgesic treatment for cancer pain.

## Methods

This study was approved by the Institutional Review Board of the University of Pennsylvania. Informed consent was obtained from all participants. A 3-month prospective observational study was conducted with repeated measures at two time-points, i.e., at baseline (T1) and 3-months (T2). Patients were recruited from two outpatient medical oncology clinics within the University of Pennsylvania Health System. Inclusion was based on self-identified African-Americans and Whites, at least 18 years of age, diagnosed with solid tumors or multiple myeloma, with cancer-related pain, and at least one prescription of around-the-clock pain medication. A trained research assistant made home visits to gather data at T1 and T2 at a time convenient for the patients.

### Conjoint analysis methodology

The CBC study was designed in consultation with Sawtooth Technologies, Inc. CBC is one of the methods within the expanding repertoire of conjoint analysis techniques. It uses a decompositional design to observe consumer choices based on how they react to a series of changes in attribute levels of a good. The main advantage of CBC is that it presents choice questions in full-profile, i.e., all attributes are presented to the respondent at one time, allowing respondents to make trade-offs between attribute levels closely mimicking how real life decisions are made [7].

The International Society for Pharmacoeconomics and Outcomes Research (ISPOR)’s *Good Research Practices for Conjoint Analysis Task Force* has recently published guidelines for the application of conjoint analysis in health [5]. Designing a CBC study involves systematic steps [18,19]. Key design elements include: Selection of attributes and levels that define the profiles in conjoint analysis tasks; construction of tasks; experimental design; and statistical analysis [18].

#### Attributes and levels

In our study, the construct of interest was *preferences for analgesic treatment for cancer pain*. Our interest was not to identify preferences for specific analgesics but most salient considerations patients have in using analgesia for cancer pain. Two constraints guided the identification of attributes: first, inclusion of the most salient attributes to minimize respondent burden and second, operationalization of attribute levels that are plausible. Both literature review and qualitative groundwork can serve to identify relevant attributes and levels [19]. In our study, separate qualitative focus groups with African-Americans and Whites [20] suggested six attributes that mattered the most to patients in considering analgesics for cancer pain treatment: 1) type of analgesic, 2) percent pain relief with analgesics, 3) type of side-effects, 4) severity of side-effects, 5) out-of-pocket cost) and 6) analgesic-related beliefs.

Of the 6 identified attributes, 5 allowed operationalization into concrete levels (i.e., type of analgesic; degree of pain relief with analgesics; type of side-effects; severity of side-effects; and out-of-pocket cost). The attribute of “analgesic-related beliefs” was excluded because this attribute is endogenous to the respondent, and cannot be varied [5]. For instance, defining beliefs as “presence or absence of beliefs” or “strong” or “weak” beliefs was not meaningful. Thus, the research team, in consultation with Sawtooth Technologies, decided to study analgesic beliefs using a different discrete choice, trade-off technique, Maximum Difference Scaling (MaxDiff) analysis. MaxDiff is a paired comparison in which respondents are asked to choose from a given set of beliefs those that are “most” and “least” important in deriving patients’ analgesic use [21]. Choices are varied systematically to understand which beliefs may underlie patients’ decision-making to use analgesia (findings are presented as part of a separate paper in review).

#### Construction of tasks

Based on the final set of attributes, a computer-assisted, CBC experiment was developed. Efficient randomized design algorithms were used in the creation of choice profiles to yield unbiased estimates of participants’ preferences. A heuristic optimization algorithm was applied using a balanced factorial design that has near perfect orthogonality (principle of independence, i.e., by varying variables individually, one can predict the combined effect of varying them jointly). This design was blocked into groups of 14 CBC tasks, with 2 treatment alternatives per task that were unique for each respondent. This study used CBC’s Complete Enumeration task generation method, which forces alternatives within each task to be kept as different as possible (minimal overlap). The computer-assisted design was flexible, efficient, and robust to response ordering effects [22,23]. The randomized design permitted data to be aggregated question-by-question allowing an examination of how utilities changed as respondents progressed through the interview. Further, the algorithms minimized the response burden, while yielding preference weights that have the smallest standard errors for the calculated sample size and design complexity.

The survey was field-tested with two separate groups of African-Americans and Whites with cancer-related pain (N = 13; African-Americans = 7, Whites = 6) to determine the comprehension and ease of completion of computer-based CBC exercise. Of note, we included patients based on a range of computer literacy *(‘*extremely comfortable’ to ‘not comfortable at all’). The investigator and a trained research assistant were available to assist patients with limited computer literacy. The final survey was modified based participants’ suggestions for improving the instructions.

#### Experimental design

The T1 and T2 CBC designs were identical. Each design consisted of 12 random tasks and *2 holdout tasks* (5^th^ and 10^th^ tasks), for a total of 14 tasks. Two treatment alternatives were displayed per task. The holdout tasks were constructed so there would be a clearly preferred, but not overly dominant alternative in each task.

The final sample size was based on concepts of power calculation that are unique to CBC design. The conventional power calculations are not applicable in CBC studies; rather, conjoint analysis experts either apply rules of thumb or past experience in determining an appropriate sample size [24]. In our study, the final sample size was based on the past experience; we use the expected standard error of 0.05 recommended by Sawtooth Software, so our results could be compared more easily with the results of other studies that use CBC. The sample requirements were based on the number of attributes, the maximum number of levels per attribute, and the effects to be measured. A larger number of attributes provide more information on trade-offs but also encourage participants to simplify heuristics due to increased task complexity [24]. Similarly, more levels per attribute provide increased “preference granularity”, but also increase the need for sample size to allow estimation of additional parameters [24].

The sample size was generated using a computer simulation that used random dummy respondent data for the specified CBC design using aggregate logit for estimating utilities. Also taken into consideration is the ability of respondents to reliably answer 10 to 20 CBC questions [23,25]. The sample size and the number of questions were varied systematically until the expected standard error associated with the utility estimates was 0.05 or smaller. This yielded an estimate of 200 subjects. The model estimated was based on main effects. Further, we enrolled a total of 240 patients to account for a projected attrition rate of 20% due to advanced disease, death, and withdrawal for other reasons. Our sample size (n = 200) exceeded the sample size requirement using Johnson’s Rule of Thumb, which recommends a minimum sample size of 125 for 2 choice alternatives; 12 task repetitions; and maximum number of 6 levels for any one attribute [24].

#### Statistical analysis

All analyses were conducted using Sawtooth Software CBC/HB system [26]. Hierarchical Bayes (HB), allowed estimation of individual-level utilities using choice data. HB borrows information from every respondent in the dataset to improve the accuracy and stability of each individual’s preference weights [7]. The relative importance and preference weights of specific analgesic attributes in determining the overall utility was analyzed using a random utility model which allowed analysis of clustered data (e.g., repeated measurements from multiple responses obtained from the same individual in this study). The function to be estimated was of the form:

$$V_{i}=X_{i}\beta +e+u$$

Where, V_i_ = overall utility or preference associated with analgesic treatment i; X_i_ = row vector of attribute-level codes representing alternative i, β = vector of part worth utilities, *e* = error that accounts for the differences amongst observations (measurement error), and *u* = error that accounts for differences amongst respondents. A utility function that monotonically increases indicates that as the level of an attribute increases so does the individual’s preference associated with that attribute. The converse is true for a utility function that monotonically decreases. The estimated utilities indicate the relative impact of different attribute levels on pain treatment decisions. The greater the relative size of the utility the greater the impact of the different *attribute levels* in determining the overall utility value.

##### Methods for internal and external predictive validity of the CBC exercise

Two holdout tasks that looked exactly like the CBC scenarios were embedded in 14 CBC tasks (one early in the survey and one late in the survey). The holdout tasks are not used in the measurement of preference weights but they provide essential insights into the validity and stability and of CBC responses [27]. The internal and external predictive validity of the CBC tasks was estimated using Root Likelihood (RLH) and Mean Absolute Error (MAE), respectively.

The RLH measures the goodness of fit between the estimated utilities and the respondent’s choice data. We calculated the likelihood of each respondent choosing as he/she did on each task, by applying a logit model using estimates of the respondent’s utilities. To compute RLH we multiplied these likelihoods together and took *n*^*th*^ root of the likelihood, where *n* is the total number of choices the respondent made. RHL is therefore the geometric mean of the predicted probabilities. If there were k alternatives in each choice task and we had no information about a respondent’s utilities, we would predict that each alternative would be chosen with probability *1/k*, and the corresponding RLH would be *1/k*. RLH would be one if the fit were perfect.

The MAE is an average of the absolute differences between the choice model predictions and the aggregate choices respondents made for two holdout tasks included in the choice exercise. The logit exponent of the model was tuned for each racial group so as to minimize the MAE for that group*.*

##### Methods for inter-temporal stability of the CBC exercise

The inter-temporal stability of the CBC was assessed using utilities from baseline and 3-months. The scores from each of the two assessment points were ranked from highest to lowest in terms of the percentage of overall importance assigned by the CBC utilities. Using Cohen’s kappa statistic, the agreement among scores across the two time-points was assessed overall and individually within each CBC attribute. We used Cohen's kappa as it is thought to be a more robust measure than the simple percent agreement calculation since kappa takes into account the agreement occurring by chance. Based on magnitude guidelines, kappa values of 0 indicates no agreement, 0–.20 as slight, .21–.40 as fair, .41–.60 as moderate, .61–.80 as substantial, and .81–1 as almost perfect agreement [28]. The agreement among the repeated assessment was also evaluated via the Spearman correlation coefficient, and an exploratory analysis to identify subgroups with strong retest characteristics.

Further, we ran a number of analyses to determine whether a very strong preference at baseline remains consistently strong at 3-months. Strong preference at baseline was defined as a ‘relative importance’ ranking for an attribute of 50% or more. Thus, a zero or one was assigned to each of the five CBC attributes for each subject based on whether the strong preference definition was met. If an attribute with the strongest preference also ranked highest or second highest at T2, the event was classified as ‘stable’, otherwise it was classified as ‘not stable’. Logistic regression was employed using the SAS [29] software package to test for statistically significant associations between having a strong preference and potential demographic and clinical variables. Summary statistics and cross-tabulations were generated to further assess associations that were significant at the 0.05 level.

##### Methods to identify predictors of inter-temporal stability

We examined whether differences in inter-temporal stability can be explained by baseline demographic (education, income, race, age, marital status, and computer literacy) and clinical variables (general health and pain levels) collected at T1. For these set of analyses, we considered both the “actual difference” and the “absolute value of the difference” in the *relative importance* scores of an attribute *within a patient* from T1 to T2 as outcome measure of inter-temporal stability. The actual change is the mathematical subtraction of T2 – T1, where T2 is the CBC relative importance score at T2, and T1 is the CBC relative importance score at T1 (for example, if T2 = 0.49 and T1 = 0.54, then the actual change would be −0.05, but the absolute change would be 0.05). The *actual difference* was generated to test for a directional association and the *absolute value of the difference* was computed to test whether any of the baseline variables predict the general variability in the inter-temporal stability over time. Thus, the T1 to T2 differences at or near zero indicated better stability. We then performed several analyses to test for significant associations among all 5 CBC attributes and baseline demographic and clinical variables.

##### Methods to identify change in information set from T1 to T2

Economic theory assumes that preferences are stable although a person’s information set may change over time. Thus, we examined several patient-specific characteristics (general health, pain levels, pain-related interference, analgesic beliefs, and social support) for evidence of a statistically significant change from T1 to T2. All of the variables considered were continuous or on a likert scale. To determine what changed *within* the patient between T1 and T2 in terms of these factors, a paired T-test, or the Wilcoxon sign rank test was used depending on the symmetry of the distribution of the difference measure (T2 minus T1). If significant changes were detected, those changes were correlated with the changes in the *relative importance* scores of the 5 CBC attributes from T1 to T2, using the Pearson and Spearman correlation coefficient.

##### Methods to identify systematic differences in those who completed both surveys and those who completed T1 only

A comprehensive set of analyses was conducted to examine whether there were any systematic differences in the baseline characteristics between *those who completed both surveys and those who only completed the survey at T1*. We began by performing a preliminary analysis of all outcomes (the 5 CBC attributes) and baseline demographic variables and tested for differences in baseline measures between the group who only provided T1 data and those who provided both T1 and T2 data. The *test for differential dropout* was employed to see if the baseline variables were equally distributed between the two groups. These baseline comparisons were based on T-tests or Wilcoxon rank sum tests for continuous variables, depending on the symmetry of the distributions and on Chi-square or Fisher’s Exact test depending on the cell sizes for binary or ordinal variables.

## Results

A total of 241 patients (African-Americans =102; Whites =139) completed the CBC survey at baseline. There were significant differences between African-Americans and Whites on a number of demographic variables including gender, marital status, education, income, and insurance status (Table 1). The attrition rate from T1 to T2 was 17% (N = 201). There was no evidence of differential dropout by key variables such as race (p = 0.496) and general health status (p = 0.612). The *relative importance* of the CBC choice structure remained stable from T1 to T2 (Table 2). The most important analgesic preference attributes at both time-points in descending order were 1) percent pain relief with analgesics, 2) type of analgesic side-effects 3) severity of side-effects 4) type of analgesics, and 5) out-of-pocket cost. At both time-points, Whites overwhelmingly traded based on “degree of pain relief” whereas for African-Americans , the most important factors in pain treatment were “type of analgesic side-effects” and “degree of pain relief” with analgesics (Table 3).

**Table 1:** Demographic characteristics of study participants by race (N = 241)

**Variable**	**Total (N = 241)**	**African-Americans (N = 102)**	**Whites (N = 139)**	**p-values†**
**Mean (SD)**				
Age	53.7 (11.0)	52.7 (10.1)	54.5 (11.6)	.194
Heath literacy score	13.0 (2.6)	13.0 (3.0)	13.0 (2.4)	.934
Comfort with computers	3.3 (1.4)	3.1 (1.4)	3.5 (1.4)	.033
**Frequency (%)**				
Gender				.019
Male	111 (46)	38 (37)	73 (53)	
Female	130 (54)	64 (63)	66 (47)	
Marital status				<.001
Married	133 (55)	33(32)	100 (72)	
Separated/ divorced/widowed	62 (26)	42 (41)	20 (14)	
Never married	46 (19)	27(27)	19 (14)	
Education				.011
Elementary	3 (1)	2 (2)	1 (2)	
High school	84 (35)	42 (41)	42 (42)	
College/trade school	117 (49)	51 (50)	66 (51)	
More than college	37 (15)	7 (7)	30 (7)	
Income				<.001
< 30, 000	85 (35)	57 (56)	28 (20)	
30-50,000	44 (18)	26 (25)	18 (13)	
50-70,000	41 (17)	13 (13)	28 (20)	
70-90,000	25 (11)	3 (3)	22 (16)	
>90,000	46 (19)	3 (3)	43 (31)	
Health insurance				<.001
Private	123 (51)	30 (29)	93 (67)	
Medicaid	33 (14)	28 (27)	5 (4)	
Medicare	50 (21)	25 (25)	25 (18)	
Multiple	26 (11)	13 (13)	13 (9)	
Other	8 (3)	6 (6)	2 (1)	
Cancer type				.895
Lung	39 (16)	14 (14)	25 (18)	
Breast	40 (17)	19 (19)	21 (15)	
GI	41 (17)	15 (15)	26 (19)	
GU/reproductive	28 (11)	12 (12)	16 (11)	
Multiple myeloma	36 (15)	17 (16)	19 (14)	
Other solid tumors	57 (24)	25 (24)	32 (23)	

†p-values are based on t-tests for continuous variables and chi-squared for categorical variables.

**Table 2:** Aggregate results of analgesic treatment utilities over-time estimated using CBC

		**At baseline (N = 241)**		**At 3-months (N = 201)**	
Treatment attributes*	**Levels**	**‡Relative importance**	**†Preference weights**	**‡Relative importance**	**†Preference weights**
**% Pain relief with analgesics**		**32.53**		**32.11**	
	< 50%		−90.3		−91.8
	60−70%		−12.9		−13.8
	80−90%		30.9		36.9
	100%		72.3		68.7
**Type of analgesic side-effects**		**23.28**		**19.71**	
	Confusion		−37.8		−23.4
	Drowsiness/Dizziness		23.8		12.0
	Heartburn/Sour Stomach		13.7		9.2
	Constipation		16.9		19.1
	Nausea/Vomiting		−16.7		−16.9
**Severity of side-effects**		**17.81**		**17.16**	
	Mild		32.5		34.1
	Moderate		24.1		17.6
	Severe		−56.6		−51.7
**Type of analgesic**		**14.85**		**15.86**	
	Oxycontin or Morphine-like		19.5		19.2
	Motrin or Aleve-like		−19.5		−19.2
**Out-of-pocket cost**		**11.53**		**15.17**	
	$10−$20		24.5		28.7
	$30−$40		20.8		18.3
	$50−$60		−0.16		6.5
	$70−$90		−11.9		−6.4
	$100 or more		−33.1		−47.1

*The attributes were identified based on pilot work with African Americans and Whites with cancer pain. ‡Relative importance scores sum to 100 across all attributes; †Aggregate utilities associated with each level of the attribute; smaller or more negative preference weight indicates less preference for that level of an attribute.

**Table 3:** Aggregate utilities of analgesic treatment for cancer pain "by race" over-time

	**At baseline (N = 241)**			**At 3-months (N = 201)**		
**Attributes & levels†**	**Whites (N = 139)**	**African-Americans (N = 102)**	**p-values**	**Whites (N = 114)**	**African-Americans (N = 87)**	**p-values**
**% Pain relief with analgesics**	**36.71**^**‡**^	**26.83**^**‡**^	**<0.001**	**35.65**^**‡**^	**27.45**^**‡**^	**.006**
< 50%	−102.5	−73.8	.000	−101.7	−78.8	.006
60-70%	−15.7	−9.12	.002	−15.2	−11.9	.337
80-90%	37.1	22.6	.000	40.4	32.3	.062
100%	81.1	60.3	.000	76.5	58.5	.007
**Type of analgesic side-effects**	**19.29**^**‡**^	**28.72**^**‡**^	**<0.001**	**16.96**^**‡**^	**23.30**^**‡**^	**.009**
Confusion	−32.1	−45.5	.081	−11.7	−38.8	.000
Drowsiness/dizziness	21.0	27.6	.145	12.8	11.1	.717
Heartburn/sour stomach	13.3	14.4	.865	7.8	11.0	.518
Constipation	18.9	14.2	.448	13.5	26.5	.047
Nausea/vomiting	−21.1	−10.7	.056	−22.4	−9.8	.048
**Severity of side-effects**	**18.55**^**‡**^	**16.81**^**‡**^	**.225**	**19.03**^**‡**^	**14.71**^**‡**^	**.027**
Mild	33.0	31.8	.636	38.2	28.7	.022
Moderate	26.7	20.6	.010	18.7	16.1	.212
Severe	−59.7	−52.3	.112	−56.9	−44.8	.034
**Type of analgesic**	**13.52**^‡^	**16.66**^‡^	.176	**14.83**^‡^	**17.19**^‡^	**.413**
Oxycontin or morphine-like pain medicine	19.9	19.0	.904	23.4	13.8	.272
Motrin or aleve-like pain medicine	−19.9	−19.0	.904	−23.4	−13.8	.272
**Out of pocket cost**	**11.93**^‡^	**10.98**^‡^	.355	**13.51**^‡^	**17.33**^‡^	**.017**
$10-$20	25.0	23.8	.608	24.7	33.9	.003
$30-$40	22.0	19.1	.122	16.1	21.2	.019
$50-$60	-.11	-.23	.889	7.9	4.6	.023
$70-$90	−12.3	−11.5	.598	−5.9	−7.1	.466
$100 or more	−34.6	−31.1	.232	−42.8	−52.7	.037

†Aggregate utilities associated with each level of the attribute; smaller or more negative preference weight indicates less preference for that level of an attribute.

‡Relative importance scores sum to 100 across all attributes. P-values are based on 2-tailed two sample t-test.

Subsequent analyses revealed that Whites had 2.8 times the odds of ranking strongest on the “percent pain relief with analgesics ” attribute than African-Americans (95% CI, 1.4 - 5.5; p = 0.002). Those with higher income tended to be more likely to rank the “percent pain relief with analgesics” CBC attribute highest at T1 (p = 0.009). Lastly, those with higher education levels also tended to be more likely to rank the “percent pain relief with analgesics” attribute highest at T1 (p = 0.007).

### Comparative validity

Overall, the internal and external predictive validity of the CBC was comparable between African-Americans and Whites. At both time-points, the internal predictive validity (RLH) of the CBC was slightly higher for Whites than for African-Americans (Table 4). The internal predictive validity for African-Americans improved at T2 nearly catching up to Whites possibly relating to learning effect (Table 4).

**Table 4:** Internal and external predictive validity of CBC utilities by race over-time

	**Internal predictive validity**				**External predictive validity**	
	**†RLH (baseline)**	**95% CI**	**†RLH (3-months)**	**95% CI**	**‡MAE (baseline)**	**‡MAE (3 months)**
**African-Americans**	0.789	0.767−0.812	0.826	0.804−0.848	3.04%	8.04%
**Whites**	0.849	0.835−0.863	0.846	0.828−0.865	4.20%	10.24%

†RLH = Root Liklihood; ‡MAE = Mean Absolute Error.

If we define dominant or lexicographic behavior in the data as having an RLH value greater than .750 (1.5 times chance) and a single attribute importance greater than 50%, then this type of behavior was observed in 29% of the choice data sets. Of these data sets, 60% were for Whites and 40% were for African-Americans.

As for external predictive validity (MAE), the CBC model was slightly better at predicting African-Americans’ choices than for Whites (MAE = 3.04% for African-Americans and 4.02% for Whites). At T2, the MAE increased for both groups i.e., 8.04% for African-Americans; 10.24% for Whites (MAE of 0 represents perfect agreement between the model and the aggregate choice data). The external validity of the T2 exercise again showed the model being slightly better at predicting African-Americans’ choices (Table 4).

#### Test for scale heterogeneity

Aggregate scale factors were estimated for each subgroup in the process of minimizing the MAEs (“tuning the choice model”). At T1, scale factors were larger for African-Americans (2.5) than for Whites (0.4), and nearly the reverse at T2 (African-Americans = 0.3; Whites = 2.5). This indicates that on average, Whites’ choices became less random from T1 to T2, whereas African-Americans’ choices became more random. This suggests that, to the extent the differences between scale factors within and between T1 and T2 are significant, experience with pain treatment may have clarified the choices for Whites, but made them more difficult for African-Americans.

### Inter-temporal stability

Inter-temporal stability was measured for each participant who had data available at both time-points. The Inter-temporal stability of CBC utilities over the 3- month time period yielded a Kappa of 0.28 with a 95% confidence interval of 0.24 to 0.32. The correlation between T1 and T2 rankings as measured by the Spearman correlation coefficient was consistent with the Kappa (Spearman Coefficient = 0.37). While in general there were differences between the T1 and T2 CBC utilities, there were subgroups that showed consistency from T1 to T2. Specifically, 88% of those who ranked the attribute of “percent pain relief” highest (defined as *relative importance* score of at least 50%) at T1, also ranked the “percent pain relief” high (1^st^ or 2^nd^) during T2; 77% of those who ranked the “analgesic side-effects” attribute highest during T1, also ranked this attribute high (1^st^ or 2^nd^) during T2; and 71% of those who ranked the “type of analgesic” attribute highest during T1, also ranked the “type of analgesic” high (1^st^ or 2^nd^) during T2 (Table 5).

**Table 5:** Inter-temporal stability of CBC utilities over-time

	**Overall**			**African-Americans**		**Whites**	
Dominant utility^*^	**Sensitivity/ specificity**^**†**^	**Kappa (95% CI)**	**Odds ratio**^**‡ **^**(95% CI)**	**Sensitivity/ specificity**^**†**^	**Kappa**^**‡ **^**(95% CI)**	**Sensitivity/ specificity**^**†**^	**Kappa (95% CI)**
**% Relief with analgesics**	0.88/0.40	0.17 (0.08, 0.25)	4.6 (1.9, 11.6)	0.62/0.46	0.04 (−0.10, 0.17)	0.97/0.34	0.22 (0.12, 0.33)
**Side-effects type**	0.78/0.59	0.13 (0.04, 0.22)	5.0 (1.6, 15.9)	0.86/0.58	0.24 (0.08, 0.39)	0.50/0.60	0.02 (−0.07, 0.10)
**Type of analgesic**	0.71/0.80	0.32 (0.17, 0.46)	10.0 (3.6, 27.6)	0.80/0.77	0.33 (0.13, 0.54)	0.64/0.83	0.29 (0.08, 0.51)

*Dominant Utility is defined as a relative importance ranking of an attribute at T1 (baseline) of at least 50%.

^**†**^Sensitivity is defined as the proportion of participants who ranked the same attribute high (either 1^st^ or 2^nd^) at T2 (3-months) as the dominant attribute at Time 1; Specificity is defined as the proportion of participants who *did not* rank the given attribute high (either 1^st^ or 2^nd^) at T2 of those who did not rank the given utility as dominant at T1.

^**‡**^The odds of the given utility to be ranked high (either 1^st^ or 2^nd^) at T2 if it is the dominant utility at T1.

Note: Very few participants had strong preferences associated with “out-of-pocket cost” and “severity of side-effects”, thus there was not enough data to evaluate the Inter-temporal stability of those preferences.

#### Predictors of inter-temporal stability

We found no statistically significant associations (at the alpha = 0.05 level) between differences in inter-temporal stability and any of the baseline (education, income, race, age, marital status, and computer literacy) and clinical variables (general health and pain levels). The Pearson and Spearman correlation coefficients ranged between −0.2 and 0.2 for all of the continuous baseline predictor variables. This was the case, regardless of whether inter-temporal stability was measured as the actual change or the absolute change from T1 to T2.

#### Change in information set from T1 to T2

We identified a number of patient-specific variables that exhibited statistically significant change from T1 to T2. These included “physical health” in past 30 days (p = 0.03); “worst pain” level (p = 0.002) and pain-related “functional interference" (p = 0.015) measured using Brief Pain Inventory [30]; and “pain management barriers” (harmful effects, p = 0.048; physiological effects, p = 0.029; and total number of pain related barriers, p = 0.017) measured using Barriers’ Questionnaire [31]. Of note, while the above mentioned patient-specific variables changed significantly over the 3 month period, none of the changes were found to be correlated with changes in the patient preferences as measured by changes in the *relative importance scores* from T1 to T2. All of the Pearson and Spearman correlations were well within −0.2 and 0.2.

#### Systematic differences in those who completed both surveys and those who completed T1 only

We found no statistically significant systematic differences in baseline demographics [age (p = 0.9052), gender (p = 0.0579), race (p = 0.5998), education (p = 0.9835), health literacy (p = 0.3317), income (p = 0.0522), type of insurance (p = 0.0631), social support (p = .2157)] and clinical characteristics [general health (p = 0.9214), average pain levels (p = 0.7256), pain-related functional interference (p = .4318), and reported analgesic barriers (p = 0.7555)] between those who completed both surveys and those who completed the survey at T1 only.

## Discussion

Widespread and concerning disparities have been documented in a variety of clinical outcomes in the U.S, although sources of disparities are not adequately explained. We continue to remain deficient in effective methods to understand sources of disparities. Conjoint Analysis can serve as an important tool to understand what unique factors may underlie decision-making of diverse subgroups, although methodological advancements are needed in exploiting this application of CA. In this study, we compared the predictive validity and temporal stability of CBC in eliciting preferences for cancer pain treatment between African-Americans and Whites.

Despite 3-month duration between the baseline and subsequent assessment, we found that the “overall” choice structures (relative importance and preference weights) remained stable from T1 to T2 (Table 2). Also, the most salient factors for pain treatment decision-making remained stable for both African-Americans and Whites (Table 3). The values of estimated utilities conformed to logic and *a priori* assumptions. For instance, for both groups, the estimated values became more negative as the attribute levels became less favorable, (e.g. more cost or less relief) (Table 3). Interestingly, for Whites expectation of “pain relief” was the more salient and consistent factor, whereas African-Americans traded between “side-effects” and “pain relief” suggesting unique concerns underlying analgesic taking behavior and possibly disparate clinical management of pain and side-effects between the two groups.

### Internal predictive validity

At both time-points, the internal predictive validity (RLH) of the CBC was slightly higher for Whites than for African-Americans (Table 4). This indicates that Whites were slightly more consistent than African-Americans when *expressing* their choices as indicated by their higher RLH (Table 4). This may reflect a higher level of involvement by Whites in the pain treatment process prior to T1 (e.g., reading up on pain treatment alternatives, doctor consultations, or participation in cancer support groups, etc.). This also suggests that Whites used lexicographic decision rules more often than African-Americans when choosing pain treatments alternatives.

In our study, lexicographic behavior was observed in 29% of choice data sets. Within this group, Whites were more likely than African-Americans to engage in a lexicographic or dominant behavior (60% vs. 40%). Lexicographic preferences occur when only one attribute matters to the individuals in considering a good or service resulting in unwillingness to trade more or less of one attribute in favor or detriment of the other. Our study provided evidence that on average Whites were significantly more likely to trade based on expected pain relief from analgesics than any other attribute (see Table 3). Lexicographic preferences may arise both from complexity of the conjoint choices but also an individual’s past experiences or expectations [32].

While we found evidence of lexicographic decision behavior in the data, we cannot determine whether it reflects actual preferences or simplifying processes since we did not debrief patients about how they made their choices. However, the concern about lexicographic preferences arising from mental shortcuts is mitigated in studies where participants view the task as relevant to their condition and thus are highly motivated to answer the questions [33]. For instance, in our study all patients had cancer-related pain and they may view certain levels of attributes as most salient or a “must” in pain treatment decision-making.

The fact that internal predictive validity for African-Americans and Whites became almost similar at T2 (Table 4) may indicate that the relative proportion of error in prediction for African-Americans at T1 may relate to learning effect (processing and evaluating the full-profile choice). Allowing participants to become familiar with CBC exercise, e.g. by incorporating mock CBC questions early on, may overcome learning bias and improve estimation of utilities.

### External predictive validity

Based on our findings on the external validity, we found that it was easier to *predict* the pain treatment decisions for African-Americans than for Whites as indicated by the smaller MAE for African-Americans at both time-points (Table 4). One likely explanation for this difference is that the two CBC holdout tasks used in determining the MAE might have presented alternative pain treatment scenarios that were more differentiated (with respect to preference system) for African-Americans than for Whites. Further, the MAE increased from T1 to T2 for both Whites and African-Americans possibly reflecting an increase in the complexity of making pain treatment decisions based on *expectations* (T1) as opposed to *reality* (T2).

### Inter-temporal stability

Based on the magnitude guidelines for kappa values, we found evidence of fair inter-temporal stability (0.28, 95% confidence interval = 0.24 to 0.32) for the CBC attributes over time. While the inter-temporal stability was only fair, this discrepancy is to be expected for a number of reasons: (1) we used Hierarchical Bayes (HB) estimation procedures in computing part-worth utilities or preference weights. HB estimates an individual's scores by using the individual's data and sharing data from other respondents [26]. Since the pool of respondents differed between T1 and T2 we would expect the individual scores to be different. This may specially affect stability of utilities for those with marginal preferences for an attribute. This is plausible since when we partitioned our analysis for only those with a high *relative importance* score of 50% or more on an attribute at T1, we found that a high percentage of individuals was likely to maintain their preferences at T2 (see Table 5); (2) one source of difference between time-points could be attributable to differences in the set of choice questions since a patient did not answer the same set of choice questions from the randomized block design at both time-points; (3) conjoint studies can be cognitively challenging and learning effects are well known; based on our findings of internal predictive validity (RLH; Table 4), learning almost certainly occurred between T1 and T2 possibly resulting in observed differences in temporal stability; (4) from a conceptual perspective, preferences may evolve overtime as a result of change in information set; a lack of stability could be considered good if it captures an individual’s real life experiences. For example, one might prefer pain relief going into treatment but prefer fewer side effects after experiencing treatment for three months. However, based on the analysis conducted, while we identified a number of relevant clinical variables that exhibited a statistically significant change from T1 to T2, none of the changes were found to be correlated with changes in the patient preferences from T1 to T2. Thus, at least based on the variables tested, change in information set could not explain the observed inter-temporal stability from T1 to T2.

### Study limitations

The findings of our study should be interpreted in the light of several limitations. In this paper, we presented a single empirical example of how predictive validity tests could be conducted as part of a CBC experiment. We estimated separate models to evaluate the performance of African-Americans and Whites on the predictive validity tests. However, estimating separate models is only one way to estimate systematic differences in preferences between groups. Alternative approaches include interaction models, nested models, and latent class models. Further, this study used CBC’s Complete Enumeration task generation method, which forces alternatives within each task to be kept as different as possible (minimal overlap). That, together with showing only two alternatives per task, also increased the likelihood of patients using lexicographic-type decision rules. Further, our sample was limited to cancer patients from one health system. Findings may vary in patients in other contexts.

## Conclusions

The analysis presented in this paper pertained to methodological issues; our goal was to assess how African-Americans and Whites performed on a systematically designed CBC experiment. More specifically we sought to compare internal and external predictive validity and inter-temporal stability of CBC in these two groups. Based on the comparative validity findings, we conclude despite slight group differences, overall the internal and external predictive validity of CBC was comparable between African-Americans and Whites. For internal validity, we found that a learning bias may have operated more so for African-Americans. Allowing participants to become familiar with CBC exercise, e.g., by incorporating mock CBC questions early on, may improve estimation of utilities by overcoming learning bias. Dominant (or lexicographic) behavior was observed in a minority (29%) of choice data sets. Incorporating debriefing or qualitative interviews as part of CBC exercise may provide insights into sources of dominant or lexicographic preferences. Unlike traditional instruments validated within the classical test theory paradigm, validity is not an inherent property of CBC surveys; holdout tasks may be included as part of CBC exercise as one way to study predictive validity. Validity of CBC survey is also based on pragmatic issues such as task complexity (e.g., number of attributes, number of levels per attribute, and number of tasks per respondent) and task relevance (e.g., how plausible attributes and levels are within a given context). These considerations should be taken into account in designing any rigorous CBC study, including studies to elucidate clinical disparities.

## Competing interests

SHM, ALH and JChittams declare no financial or non-financial competing interests. JCurry has declared competing financial interest as the President of Sawtooth Technologies, Inc. The company routinely assists academics in the design and analysis of conjoint analysis studies. The authors declare that they have no competing interests.

## Authors’ contributions

This study was conceived by SHM. SHM contributed to all aspects of research and writing. JCurry assisted with the analysis of conjoint data as well as offered methodological input during all the stages of research. ALH and JChittams assisted with the analysis of the temporal stability aim of the study. Writing of the manuscript was led by SHM and all authors provided critical feedback and approved the final version.

## Pre-publication history

The pre-publication history for this paper can be accessed here:

http://www.biomedcentral.com/1472-6947/13/118/prepub
